# Clonal Hematopoiesis of Indeterminate Potential (CHIP): A Model of Mutation-Driven Thromboinflammation

**DOI:** 10.3390/cancers18091326

**Published:** 2026-04-22

**Authors:** Bouse Malkots, Iliana Stamatiou, Emmanuil Panagiotopoulos, Lydia Inglezou, Vasiliki Sakka, Georgios Vrachiolias, Christina Misidou, Emmanuil Spanoudakis, Ioannis Kotsianidis, Konstantinos Liapis

**Affiliations:** Department of Hematology, Democritus University of Thrace, 68 100 Alexandroupolis, Greece; iliana_o6@hotmail.com (I.S.); manolispan44@gmail.com (E.P.); lydiaiggl@gmail.com (L.I.); vasiliasakka@gmail.com (V.S.); george_vrachiolias@yahoo.com (G.V.); xmisidou@yahoo.gr (C.M.); espanoud@med.duth.gr (E.S.); ikotsian@med.duth.gr (I.K.); koliapi@med.duth.gr (K.L.)

**Keywords:** clonal hematopoiesis, CHIP, myelodysplastic syndrome, MDS, somatic mutations, thrombosis, thromboembolism, cardiovascular disease, atherosclerosis, inflammation, immunothrombosis, thromboinflammation, inflammaging

## Abstract

Clonal hematopoiesis refers to the clonal expansion of hematopoietic stem and progenitor cells, driven by somatic mutations. Major mutated genes in clonal hematopoiesis include genes involved in epigenetic regulation including DNA methylation and/or chromatin modification (e.g., *DNMT3A*, *TET2*, and *ASXL1*), tumor suppressors (e.g., *TP53*), signal transduction (e.g., *JAK2*), and RNA splicing (e.g., *SF3B1* and *SRSF2*). Clonal hematopoiesis includes clonal hematopoiesis of indeterminate potential (CHIP), clonal cytopenia of unknown significance (CCUS), and myelodysplastic syndromes/neoplasms (MDS). CHIP occurs when the frequency of the variant allele equals or exceeds 2% (4% for X-linked genes in males) in the absence of cytopenias. CHIP is common among older persons and is associated with an increased risk of hematologic cancer. CHIP is also associated with an increased risk of atherosclerotic disease including acute myocardial infarction, stroke, cardiac failure, and abdominal aneurysm. Increasing evidence suggests that CHIP is associated with venous thromboembolic disease. Somatic mutations lead to proliferation of hematopoietic progenitor cells and their progeny, resulting in excessive activation of granulocytes and monocytes. It could be postulated that chronic inflammation caused by clonal expansion of myeloid cells carrying mutations in *DNMT3A*, *TET2*, and *ASXL1* (“DTA”) genes may constitute an independent risk factor in clot formation and endothelial-cell damage. DTA mutations correlate with elevated proinflammatory cytokines such as IL-1β and IL-6 and enhanced activation of inflammasomes. Moreover, *JAK2* mutations may have a direct role in the activation of platelets and coagulation. In vivo murine studies have demonstrated that activation of the *JAK-STAT* signaling pathway promotes neutrophil extracellular trap (NET) formation, contributing to a prothrombotic state. Insights from related clonal disorders such as paroxysmal nocturnal hemoglobinuria and the VEXAS syndrome support the concept that mutation-driven innate immune activation can directly perturb hemostatic balance. This review aims to summarize the association between clonal expansion of hematopoietic cells and thrombotic disease, and highlight how somatic mutations in hematopoietic cells may contribute to vascular disease and thrombogenesis.

## Simple Summary

Clonal hematopoiesis of indeterminate potential (CHIP) is associated with increased cardiovascular morbidity and mortality. In 2014, an association between CHIP and coronary artery disease was reported, representing a major breakthrough. While its role in arterial atherosclerotic disease is well established, its contribution to venous thromboembolism (VTE) remains less clearly defined. Results of recent cohort studies suggest a modest but measurable increase in VTE risk among individuals with somatic mutations. In particular, it has been suggested that CHIP may promote chronic low-grade inflammation, endothelial activation, enhanced platelet reactivity, and dysregulated coagulation, supporting an immunothrombotic model. Thus, CHIP may serve as a genetic basis underlying otherwise unexplained thrombotic events, redefining our understanding of mutation-driven thromboinflammation.

## 1. Introduction

Clonal hematopoiesis constitutes the expansion of hematopoietic cells harboring driver mutations. Each mutation has a proportional influence on the clinical phenotype, reflecting the risk of progression to myelodysplastic syndrome/neoplasm (MDS) and acute myeloid leukemia (AML) [1]. Sequencing studies have related recurrent mutations with several types of hematologic disorders; however, similar mutations have been found in the general population, so-called clonal hematopoiesis of indeterminate potential (CHIP) [2]. The accumulation of somatic mutations in otherwise healthy human cells is common and increases steadily with age, occurring in more than 10% of people over the age of 70 years [2].

Over the past few years, evidence has revealed that age-related clonal hematopoiesis is associated with an increased incidence of cardiovascular diseases, linking specific myeloid somatic mutations to a heightened risk of atherosclerosis, myocardial infarction, stroke, and heart failure [3]. While the impact of clonal hematopoiesis on arterial disease is well established, its role in venous thromboembolism (VTE) remains less clear and warrants further investigation. Recent genetic studies have assessed the thrombotic potency of CHIP. However, most studies are limited by sample size, and the findings are considered preliminary, underscoring the need for larger, prospective analyses to determine whether CHIP truly represents an independent risk factor for VTE.

The cascade of events underlying the thrombotic propensity in CHIP awaits full elucidation. CHIP is thought to promote a state of chronic, low-grade inflammation that may represent a central mechanism underlying its diverse clinical complications, including thrombotic propensity [4]. Advances in elucidating the mechanisms of SARS-CoV-2 infection, along with intensive efforts to develop effective therapeutic and preventive strategies, have substantially deepened our understanding of the molecular pathways linking inflammation and thrombosis. Inspired by these insights, an increasing number of studies are now investigating the immunothrombotic potential of clonal driver mutations and their capacity to activate inflammatory and coagulation pathways [5,6].

By acquiring a deeper understanding of the interactions between inflammation and coagulation, we may be able to clarify the thrombotic potential of clonal hematopoiesis and identify targeted strategies for risk stratification and therapeutic intervention. In this review, we first summarize the epidemiologic evidence linking CHIP to arterial and venous thrombotic outcomes. We then examine proposed mechanisms, distinguishing CHIP-specific findings from broader evidence, and compare mutation-specific effects. Finally, we discuss related clonal disorders and the clinical implications of CHIP for risk stratification.

## 2. Clinical Significance of CHIP

CHIP is defined by the presence of acquired (somatic) mutations in hematopoietic stem cells with variant allele frequency (VAF) ≥ 2% (≥4% for X-linked genes in males) without obvious dysplastic hematopoiesis, evidence of hematologic malignancy or persistent cytopenia [1]. These mutations are mainly observed in the DTA genes (*DNMT3A*, *TET2*, and *ASXL1*) involved in epigenetic regulation, and less frequently in the *JAK2* and *TP53* genes, which are involved in hematopoietic differentiation and DNA repair, respectively [7,8]. The prevalence of CHIP increases markedly with age, reaching approximately 10% in individuals older than 70 years. This phenomenon is explained by the observation that with age advancement, the hematopoietic stem cells accumulate somatic mutations as a result of repeated cellular divisions, oxidative stress and environmental factors (smoking, chemotherapy, chronic inflammation and gut microbiota dysbiosis). In some cases, a mutation may offer a survival-growth advantage to the cell, leading to clonal expansion and clonal hematopoiesis [2,7].

Although CHIP itself does not constitute a malignancy, it is strongly associated with an increased risk of myeloid neoplasms, particularly myelodysplastic syndrome/neoplasm (MDS) and acute myeloid leukemia (AML). CHIP is increasingly recognized as a premalignant precursor state of overt myeloid neoplasia and carries an approximately 0.5–1% annual risk of developing hematologic malignancy (MDS or AML). In fact, most MDS cases are thought to have a molecularly definable “CHIP-like” precursor stage that can last for years before the development of frank myelodysplasia. In this regard, CHIP may be seen as an early step in leukemogenesis, predisposing affected individuals to an increased risk of subsequent malignant transformation [7]. Related entities such as idiopathic cytopenia of undetermined significance (ICUS) and clonal cytopenia of undetermined significance (CCUS) are also considered precursor myeloid conditions that may evolve into MDS. Importantly, the risk of progression to MDS or AML increases with clone size, as reflected by a higher VAF. The characteristics of ICUS, CHIP, CCUS, and MDS are summarized in Table 1.

Apart from its association with hematologic malignancy, CHIP has emerged as a clinically important contributor to non-hematologic disease, particularly cardiovascular pathology and is increasingly being investigated in relation to venous thromboembolism and other inflammatory conditions. Epidemiologic studies have shown that CHIP is associated with increased all-cause mortality, largely driven by excess cardiovascular mortality, as well as a markedly elevated risk of coronary heart disease, ischemic stroke, myocardial infarction, heart failure, and venous thrombosis. Importantly, this risk appears to be gene- and clone size-dependent, with larger clones and mutations in genes such as *TET2*, *DNMT3A*, *ASXL1*, and especially *JAK2* V617F conferring greater cardiovascular risk [2,7,9].

Furthermore, in patients with solid tumors, the presence of CHIP is strongly associated with an increased risk for therapy-related myeloid neoplasms (tMNs) after chemotherapy or radiation therapy, particularly in the context of high-risk mutations such as *TP53.* In addition, CHIP may contribute to adverse clinical outcomes of the primary tumor. These observations support its biological relevance beyond hematologic malignancy [10].

### 2.1. Association Between CHIP and Arterial Disease

Despite progress in the fields of diagnosis and treatment over recent decades, coronary artery disease still remains the main cause of morbidity and mortality worldwide. Factors such as hypertension, dyslipidemia, smoking, diabetes mellitus and obesity explain a main part of the cardiovascular risk; however, a significant percentage of patients present cardiovascular events without obvious “classic” factors―a phenomenon called “residual cardiovascular disease risk”. Recently, CHIP has emerged as a potential contributor to the residual cardiovascular risk. Jaiswal and colleagues, in a landmark analysis of over 17,000 individuals, demonstrated that carriers of CHIP had a 2- to 4-fold increased risk for coronary heart disease (HR 2.0; 95% CI 1.2–3.4) and ischemic stroke (HR 2.6; 95% CI 1.4–4.8) compared with noncarriers [2]. These findings were later confirmed in another case–control analysis, which showed a similar increase in coronary heart disease risk (HR 1.9; 95% CI 1.4–2.7) [3]. Notably, CHIP mutations have been identified in more than 20% of patients with myocardial infarction in the absence of traditional cardiovascular risk factors [9].

Subsequent studies have strengthened these observations and provided mechanistic insight. Analyzing large human genetic datasets, Bick et al. confirmed that carriers of CHIP—particularly those with *TET2* mutations—had increased risk of coronary artery disease; however, this excess risk was significantly attenuated in individuals harboring the IL6R loss-of-function variant (p.Asp358Ala), which reduces IL-6 signaling [11]. Vlasschaert et al. validated and expanded this outcome in a massively larger UK Biobank cohort in which CHIP was associated with an increased risk of incident coronary artery disease (HR, 1.22; 95% CI, 1.12–1.32), with even higher risk observed in individuals with larger clones, i.e., VAF ≥ 10% (HR, 1.25; 95% CI, 1.13–1.39) [12]. This excess risk was attenuated in carriers of the IL6R p.Asp358Ala polymorphism, providing human genetic evidence for a causal role of IL-6–mediated inflammation in CHIP-associated atherosclerotic disease [11,12]. Importantly, the cardiovascular consequences of CHIP do not appear to be restricted to canonical inflammatory drivers such as *TET2* and *DNMT3A*, as more recent data indicate that *TP53*-mediated clonal hematopoiesis is likewise associated with an increased risk of incident atherosclerotic disease with a HR between 1.3 and 1.4, supporting the concept that vascular risk in CHIP varies according to the underlying mutation [13]. Moreover, Bhattacharya et al. found that individuals with clonal hematopoiesis had a significantly higher risk of incident ischemic stroke compared with noncarriers (HR, 1.4–1.5), independent of traditional vascular risk factors, with the strongest association observed in carriers of *TET2* mutations [14].

However, some aspects of the clinical phenotype remain less clearly defined. In an analysis of 63,700 participants from five randomized trials of The Thrombolysis in Myocardial Infarction (TIMI) Study Group, Marston et al. found that CHIP was not significantly associated with major cardiovascular events overall (adjusted HR [aHR] 1.07), although it was associated with a higher risk of first myocardial infarction (aHR 1.31), but not recurrent myocardial infarction [15]. These findings suggest that CHIP may be more relevant to incident atherothrombotic events than to recurrent events in heavily treated populations and indicate that CHIP does not yet function as a clearly actionable biomarker for routine cardiovascular therapeutic stratification.

### 2.2. Association Between CHIP and Venous Thromboembolism

While the association of clonal hematopoiesis with cardiovascular diseases has been investigated in many studies, the role of CHIP in venous thromboembolism remains unclear. However, the impact of CHIP on venous thromboembolic events has gained significant interest in recent years. Available human studies suggest that CHIP is associated with a modest increase in venous thrombotic risk, but the magnitude of this association appears smaller and less consistent than that observed for atherosclerotic disease.

A prospective cohort study by Saadatagah et al. [16] examined whether CHIP contributes to VTE risk in older adults within the Atherosclerosis Risk in Communities (ARIC) study. Incident VTE occurred in 4.5% of individuals with CHIP compared with 3.2% of noncarriers over a median 7.1-year follow-up (HR, 1.49; 95% CI, 1.02–2.17; *p* = 0.038), with the association driven particularly by *TET2* mutations [16,17]. Although participants with hematologic cancer were excluded to reduce confounding bias, some influence from malignancy-related thrombosis could not be entirely ruled out [16]. Consistent with these findings, an independent analysis from the UK Biobank by Dikilitas et al. demonstrated that CHIP carriers also exhibited a significantly elevated risk of incident VTE (incidence rate ratio [IRR], 1.60; 95% CI, 1.04–2.46; *p* = 0.032), specifically pulmonary embolism (IRR, 1.80; 95% CI, 1.08–3.05; *p* = 0.025). Elevated risk appeared particularly driven by *TET2*-mutated CHIP, mirroring findings from the ARIC study [18].

The thrombotic risk varies substantially across CHIP–associated mutations and is highest in *JAK2* V617F mutation. More recently, a large biobank-based analysis by Zon et al. confirmed that individuals with *JAK2* V617F-mutant CHIP exhibited a substantially stronger association with VTE compared to those with other CHIP-related mutations [19]. *TET2* was associated with a significantly increased risk of incident VTE (HR, 1.33; 95% CI, 1.05–1.69; *p* = 0.02), whereas *DNMT3A* and *ASXL1* showed no measurable effect. The presence of *JAK2* V617F markedly increased the thrombotic risk, predicting both incident VTE (HR, 4.2; 95% CI, 2.18–8.08; *p* < 0.01) and prevalent VTE (odds ratio [OR], 6.58; 95% CI, 2.65–16.29; *p* < 0.01). Importantly, these associations remained robust even after excluding individuals with previously undiagnosed myeloproliferative neoplasms, with *JAK2* V617F maintaining very high-risk estimates for incident (HR, 6.24; 95% CI, 2.8–13.9; *p* < 0.01) and prevalent VTE (OR 11.88; 95% CI, 4.2–33.59; *p* < 0.01). The investigators attributed this to the prothrombotic biology of JAK2 kinase activation, which has been well established in myeloproliferative neoplasms. Their findings suggest that the *JAK2* V617F mutation represents a distinct high-risk subset within CHIP, highlighting the importance of differentiating mutation-specific thrombotic risk when evaluating CHIP in clinical or research contexts [19].

Additional observational studies further support an association between CHIP and venous thromboembolic events. Englisch et al. in a case–control study demonstrated that CHIP mutations were detected more frequently in individuals with VTE (10.3%) than in controls (3.9%) (OR, 2.74; 95% CI, 0.95–9.16), supporting the concept that clonal hematopoiesis represents an independent risk factor for venous thrombosis [20]. In a retrospective case–control study by Soudet and colleagues, sequencing peripheral blood samples from 61 patients with unprovoked pulmonary embolism revealed CHIP in 12 individuals (20%). CHIP was more frequent among patients with a history of pulmonary embolism, as compared with age-and-sex-matched general population controls [21]. In a larger study including 464,417 individuals, Liu et al. [22] observed that CHIP was associated with an increased risk of pulmonary embolism (HR, 1.17; 95% CI, 1.05–1.31) among individuals with CHIP. The increased risk was mainly noted for mutations in *TET2* (HR, 1.42; 95% CI 1.16–1.74) or *JAK2* V617F (HR, 4.17; 95% CI 2.09–8.35) whereas *DNMT3A*, *ASXL1*, *PPM1D*, and *SRSF2* mutations showed no significant effect.

Nevertheless, several uncertainties remain. The absolute effect size of CHIP on VTE risk appears modest overall, particularly for non-*JAK2* genotypes, and is substantially lower than that associated with established inherited or acquired thrombophilias [16]. In addition, some observational studies may still be susceptible to residual confounding, particularly by occult malignancy, inflammation, or undiagnosed hematologic disease. Accordingly, although the current evidence supports CHIP as a plausible host modifier of venous thrombotic risk, it is not yet sufficient to support its use as a stand-alone biomarker for VTE risk prediction or thromboprophylactic decision-making.

Taken together, the available human data support an association between CHIP and venous thromboembolic disease, but this relationship appears more heterogeneous, more genotype-dependent, and less definitively established than that observed in arterial vascular disease. This distinction is important, as it suggests that the contribution of CHIP to thrombosis is likely to depend on both the vascular context and the underlying driver mutation. Key epidemiologic studies highlighting gene-specific thrombotic risk are summarized in Table 2.

### 2.3. Clonal Hematopoiesis in Chronic Inflammatory Diseases

Recent clinical data increasingly highlight a dynamic interaction between chronic inflammation and clonal hematopoiesis, which is not unidirectional but reciprocal. It has been reported that there is a higher prevalence of CHIP in chronic inflammatory disorders such as systemic sclerosis [24], systemic lupus erythematosus [25], inflammatory bowel disease [26], and human immunodeficiency virus (HIV) infection [27,28], even among younger individuals in whom CHIP is typically rare. These observations suggest that sustained inflammatory signaling may promote the emergence or expansion of mutant hematopoietic clones beyond what would be expected on the basis of aging alone.

The inverse association between anti–TNF therapy and CHIP frequency in inflammatory bowel disease [26] further suggests that inflammatory cytokines create a selective environment favoring the expansion of mutant hematopoietic clones. Experimental studies demonstrate that inflammatory stress promotes the expansion of CHIP-mutant hematopoietic stem and progenitor cells [29,30], while mutant myeloid progeny amplify cytokine production, creating a self-perpetuating inflammatory milieu [31,32].

**Figure 1:**
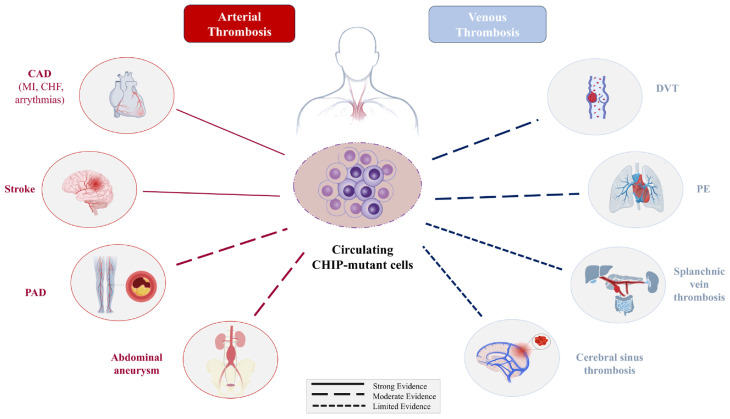
CHIP and vascular disease. Circulating CHIP-mutant cells in peripheral blood contribute to both arterial and venous thromboembolic disease. Arterial thrombotic events include cardiovascular disease (CAD) (myocardial infarction/MI, heart failure, cardiac arrhythmias), ischemic stroke, peripheral artery disease (PAD), and abdominal aortic aneurysm. Venous thrombotic events include deep vein thrombosis (DVT), pulmonary embolism (PE), and atypical venous thrombosis (splanchnic and cerebral sinus thrombosis).

Taken together, these findings support a conceptual framework in which clonal hematopoiesis and chronic inflammation exist in a reciprocal, self-reinforcing relationship: inflammatory stress favors the emergence and expansion of mutant hematopoietic clones, while these clones in turn sustain maladaptive inflammatory signaling. Within this model, CHIP may function not merely as a biomarker of aging, but as an active biological amplifier of aging-related disorders such as atherosclerosis, venous thrombosis, and solid tumors (Figure 1) [4].

### 2.4. Myeloid Malignancies at the Intersection of Cardiovascular and Thrombo-Inflammatory Risk

The clinical implications of clonal disorders-related vascular risk likely extend into overt myeloid malignancy, particularly MDS and chronic myelomonocytic leukemia (CMML), which represent advanced states along the continuum of clonal hematopoietic evolution. These disorders are increasingly understood not only as hematologic neoplasms, but also as inflammatory disease states characterized by immune dysregulation and systemic inflammatory activation [7,33]. Supporting this view, Weeks et al. [34] demonstrated in a large SEER-Medicare case–control study that patients with MDS/CMML had a significantly higher prevalence of antecedent inflammatory comorbidities—including cardiovascular and metabolic disease—than age-and sex-matched controls. In the context of thrombo-inflammatory disease, these findings are particularly relevant, as they suggest that the vascular phenotype associated with CHIP may not be confined to the premalignant stage but may remain operative, or even intensify, after progression to overt myeloid malignancy.

### 2.5. CHIP, Solid Tumor Biology, and Cancer-Associated Thrombosis

Beyond its established clinical associations, accumulating evidence suggests that CHIP may also have a more direct role in the biology of solid tumors. In this context, a recent landmark study by Pich et al. [35] introduced the concept of tumor-infiltrating clonal hematopoiesis (TI-CH), demonstrating that CHIP-derived hematopoietic clones can be detected within the tumor microenvironment of solid cancers rather than remaining confined to the peripheral circulation. In particular, *TET2*-mutant clones were found to be enriched within tumors, and functional analyses suggested that these mutant myeloid populations may actively promote tumor progression by reshaping the inflammatory and immune landscape of the tumor microenvironment.

Whether this biology also translates into an increased risk of cancer-associated thrombosis (CAT) remains uncertain. This is a clinically relevant question, given the close relationship between cancer and venous thromboembolism, and the central role of inflammation and myeloid activation in the pathogenesis of CAT. Cancer is identified in up to one-quarter of patients presenting with venous thromboembolism, and active malignancy confers an approximately 18-fold increased risk of VTE, underscoring the strong link between cancer and thrombosis [36]. However, current human data do not support CHIP as an independent major determinant of thrombotic risk in patients with solid tumors. In the first large-scale genomic analysis of molecular determinants of CAT, Dunbar et al. identified several tumor-intrinsic somatic mutations associated with thrombotic risk, whereas CHIP itself was not associated with an increased rate of VTE [37].

Cumulatively, current evidence supports a role for CHIP in shaping solid tumor biology, whereas its contribution to cancer-associated thrombosis remains unproven. At present, the strongest support lies in the concept of tumor-infiltrating CHIP, whereas any role in CAT remains speculative and requires further investigation.

## 3. Translational Evidence Linking CHIP to Thromboinflammation

The hypothesis that inflammation is a central driver of CHIP-associated vascular risk is supported not only by observational and genetic studies, but also by emerging human interventional and translational evidence. In an exploratory genomic analysis of the CANTOS trial, Svensson et al. identified CHIP in 8.6% of sequenced participants, with a predominance of TET2 mutations within this inflammation-enriched cohort [23,38]. Although CHIP carriers in the placebo arm exhibited only a nonsignificant trend toward increased major adverse cardiovascular events (MACE), patients harboring TET2-mutant CHIP derived substantial benefit from IL-1β inhibition with canakinumab [23]. These findings provide important human evidence that IL-1β–mediated inflammation is a key contributor to TET2-associated cardiovascular risk and suggest that mutation-specific subsets of CHIP may be differentially responsive to targeted anti-inflammatory strategies.

Additional translational evidence indicates that the impact of CHIP may extend beyond systemic inflammation to include direct involvement in diseased vascular tissue. In this regard, Büttner et al. demonstrated a high concordance between CHIP-associated mutations detected in peripheral blood and those identified within matched atherosclerotic plaques from patients with peripheral artery disease, with approximately 90% of mutations shared between compartments [39]. These findings support the concept that CHIP-derived clonal populations may localize to sites of vascular injury and contribute to local inflammatory processes within the arterial wall, although the potential contribution of residual blood contamination cannot be fully excluded. Overall, these observations support a model in which CHIP-associated mutations contribute to both systemic and tissue-level vascular inflammation.

## 4. Molecular and Cellular Mechanisms of Thrombosis in CHIP

Although growing clinical and experimental evidence links clonal hematopoiesis to thrombotic vascular disease, the mechanistic pathways connecting CHIP to thrombosis remain incompletely resolved. Available data support a model in which CHIP perturbs multiple components of the immunothrombotic axis, including inflammatory myeloid signaling, endothelial activation, leukocyte recruitment, platelet reactivity, and coagulation. The following sections outline the proposed mechanisms that may link CHIP to thromboinflammatory vascular disease.

### 4.1. Vascular Consequences of Inflammation

The endothelial monolayer forms the interface between blood and the vessel wall, preventing circulating coagulation factors from contacting prothrombotic extracellular matrices. Under normal conditions, the endothelium maintains vascular homeostasis and has the ability to suppress platelet activation and limit inflammation by releasing prostacyclin, nitric oxide (NO), and the ectonucleotidase CD39. Healthy endothelium also maintains a fibrinolytic balance by producing tissue-type plasminogen activator (tPA) and urokinase plasminogen activator (uPA), promoting plasmin generation and fibrin accumulation. Surface-bound anticoagulant mechanisms further reinforce this protective state [40,41]. Glycosaminoglycans (heparan sulfate, chondroitin sulfate), tissue factor pathway inhibitor (TFPI), endothelial protein C receptor (EPCR) and thrombomodulin collectively inhibit coagulation and dampen inflammatory signaling [40]. Through these mechanisms, the intact endothelium functions as a potent anticoagulant, anti-inflammatory and fibrinolytic barrier.

In case of injury or inflammation, endothelial cells shift from an anticoagulant to a proinflammatory and procoagulant state. Stimuli such as cytokines, hypoxia, disturbed flow, and bacterial endotoxins reduce endothelial anticoagulant activity and increase adhesion molecules and cytokine expression. Moreover, metabolic and inflammatory stressors (e.g., hyperglycemia or sepsis) promote degradation of the endothelial glycocalyx, eliminating heparan- and chondroitin-sulfate sites required for antithrombin III activity. Concurrent shedding of EPCR and thrombomodulin disrupts the protein C pathway, reducing anticoagulant and anti-inflammatory capacity [42,43]. Concomitantly, endothelial cells upregulate adhesion glycoproteins, including P-selectin, E-selectin, intracellular adhesion molecule-1 (ICAM-1) and vascular cell adhesion molecule-1 (VCAM-1). In particular, P-selectin is rapidly mobilized from Weibel–Palade bodies and plays a pivotal role in venous leukocyte recruitment and VTE pathogenesis [44,45]. Added to this, von Willebrand factor is also released from Weibel–Palade bodies, which enables platelet adhesion via GPIbα and promotes leukocyte recruitment under low shear, establishing a local prothrombotic niche [46].

Diverse inflammatory states, including sepsis, SARS-CoV-2 infection and hemoglobinopathies, result in endothelial cell activation through engagement of pathogen-associated molecular patterns (PAMPs), damage-associated molecular patterns (DAMPs), cytokines, and autoantibodies, followed by nuclear factor κB (NF-κB) activation, and transcription of procoagulant and proinflammatory mediators [6,47,48,49,50]. Endothelial NF-κB signaling is a key driver of vascular inflammation, as evidenced by murine atherosclerosis models in which endothelial-specific inhibition of canonical NF-κB markedly reduces Western diet–induced plaque formation [51]. By promoting adhesion molecule expression and releasing inflammatory mediators that activate and remodel smooth muscle cells, endothelial NF-κB initiates the inflammatory cascade that drives intimal thickening and progressive plaque growth [51].

Although not all of these mechanisms have been directly demonstrated in CHIP-specific models, emerging evidence suggests that clonal hematopoiesis may cause endothelial dysfunction through persistent inflammatory signaling. In this regard, studies in lower-risk myelodysplastic syndromes/neoplasms (MDS), which share features of clonal myeloid dysfunction, have identified biologically distinct inflammatory programs driven by somatic mutations, including activation of NF-κB–dependent cytokine pathways, NLRP3 inflammasome signaling, and interferon-responsive circuits [52]. In this context, myeloid-specific somatic mutations push monocyte and macrophage differentiation toward a pro-inflammatory phenotype characterized by exaggerated NF-κB signaling and enhanced transcriptional priming of the NLRP3 inflammasome, including increased expression of NLRP3, pro–IL-1β, and pro–CASP1 [53]. Even in the absence of overt injury, mutant macrophages secrete elevated IL-1β due to increased NF-κB–dependent transcription, establishing a background of persistent, low-grade inflammation that influences vascular homeostasis [52,54].

This chronic, low-grade inflammatory milieu may lower the threshold for vascular activation by priming circulating monocytes, granulocytes and platelets for exaggerated inflammasome activation when they encounter DAMPs or PAMPs. Inflammasome activation leads to increased production of IL-1β and IL-18, which drives endothelial NF-κB signaling, disrupts the anticoagulant surface phenotype, and induces the expression of adhesion molecules and tissue factor. This endothelial activation, in turn, promotes thromboinflammatory responses, including leukocyte recruitment to the activated vascular surface [40,52,55]. Within this framework, CHIP-associated inflammation may not independently initiate thrombosis but may sensitize the vascular endothelium to thromboinflammatory activation.

### 4.2. Immunothrombosis as a Mechanistic Link Between CHIP and Thrombosis

Among the available mechanistic paradigms, immunothrombosis provides the most plausible biological basis for linking clonal hematopoiesis to thrombosis. Although many of its core components were first defined in the broader contexts of venous thrombosis and vascular inflammation, this model is highly relevant to CHIP, in which persistent myeloid-driven inflammation is increasingly recognized as a central biological feature.

**Figure 2:**
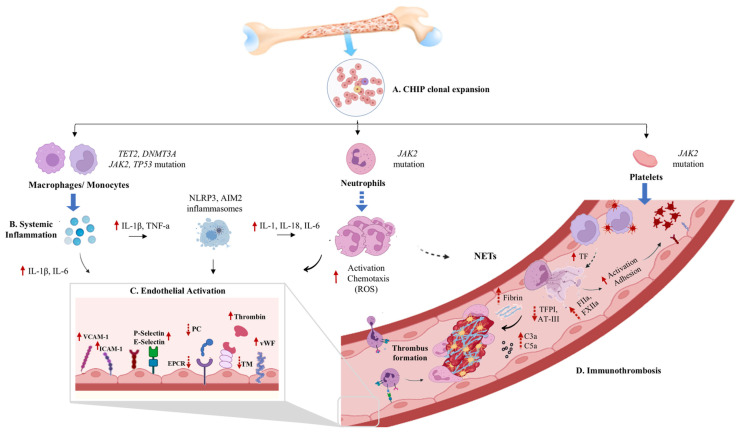
CHIP and immunothrombosis. The figure presents an integrated mechanistic model in which the strength of supporting evidence varies across illustrated steps. Solid arrows (→) indicate mechanisms directly demonstrated in CHIP carriers or CHIP-specific experimental models, dashed arrows (⇢) indicate proposed mechanisms extrapolated from related conditions or general thromboinflammatory models. (**A**) CHIP-associated mutations (*TET2*, *DNMT3A*, *JAK2*, *TP53*) drive clonal expansion of pro-inflammatory myeloid and platelet lineages [2,7]. (**B**) Mutant monocytes/macrophages exhibit enhanced inflammasome activity (NLRP3, AIM2) and increased production of proinflammatory cytokines (IL-1β, IL-6, and TNF-α), promoting systemic inflammation [52,53,54,59,60]. *TET2* mutations drive NLRP3 inflammasome activation, whereas *ASXL1* mutations preferentially activate AIM2 via cytosolic DNA damage [53,59,61]. CHIP-derived neutrophils display increased chemotaxis, ROS generation, cytokine release (IL-1, IL-18, IL-6), and NET formation. NET formation has been directly demonstrated in *JAK2*-mutant models but remains insufficiently characterized in non-*JAK2* CHIP contexts [62,63]. (**C**) Cytokine-driven endothelial upregulation of adhesion molecules (VCAM-1, ICAM-1, P-selectin, E-selectin) and vWF release are well established in inflammatory states and supported indirectly in CHIP [40,41,42,43,44,45,46]. *JAK2* V617F has been shown to directly activate endothelial P-selectin and vWF secretion [64]. Impairment of the protein C pathway via reduced TM and EPCR, facilitating leukocyte adhesion and platelet recruitment, has not been directly demonstrated in CHIP and is extrapolated from general endothelial inflammatory models [40,42,43]. (**D**) Inside the vessel lumen, NETs provide a prothrombotic scaffold for platelet adhesion and fibrin deposition [56,63]. NET-driven intrinsic coagulation activation (FXIIa, FXIa), complement activation (C3a, C5a), and inhibition of natural anticoagulants (TFPI, AT-III) are mechanistically consistent with the broader thromboinflammatory model but have not been directly demonstrated in CHIP carriers [40,50]. TF expression by activated monocytes/macrophages amplifying thrombin generation and thrombus formation is established in general immunothrombosis but remains inferred as a CHIP-specific mechanism [56]. CHIP, clonal hematopoiesis of indeterminate potential; AT-III, antithrombin III; EPCR, endothelial protein C receptor; FXI/FXII, coagulation factors XI/XII; ICAM-1, intercellular adhesion molecule-1; IL, interleukin; NET, neutrophil extracellular trap; ROS, reactive oxygen species; TF, tissue factor; TFPI, tissue factor pathway inhibitor; TM, thrombomodulin; TNF-α, tumor necrosis factor-α; VCAM-1, vascular cell adhesion molecule-1; vWF, von Willebrand factor.

Widespread endothelial cell activation underlies the multiorgan thromboinflammatory phenotype seen in acute and chronic inflammatory diseases. Activated endothelial cells facilitate rapid leukocyte recruitment to sites of disturbed blood flow, injury, or inflammation. Although leukocytes are not part of the coagulation cascade, their role in immunothrombosis is crucial. A pivotal study by von Brühl et al. demonstrated that venous thrombosis can occur on intact but activated endothelium under flow restriction [56]. In this murine DVT model, turbulent or stagnant venous flow rapidly activated the endothelial surface, enabling early adhesion of neutrophils and monocytes within hours. This early recruitment was driven primarily by P-selectin—P-selectin glycoprotein ligand-1 (PSGL-1) interactions and chemokine signaling through C-X-C motif chemokine receptor 2 (CXCR2). Once recruited to the endothelial surface, leukocytes become active drivers of thrombus development. Neutrophils promote clot formation by releasing neutrophil extracellular traps (NETs), i.e., chromatin structures that are loaded with tissue factor and other clotting factors and create a framework that facilitates platelet adhesion and fibrin accumulation. Monocytes contribute by serving as a major source of tissue factor, thereby maintaining activation of the extrinsic coagulation pathway and continuous thrombin production. Platelets may enhance these processes by attaching to NETs, secreting procoagulant substances, and reinforcing the local inflammatory milieu [56]. In addition, platelets and myeloid cells release extracellular vesicles enriched in procoagulant and inflammatory mediators, facilitating thrombus propagation both locally and at distant sites. Consistent with these cooperative interactions, inhibition of leukocyte adhesion molecules―particularly P-selectin and PSGL-1―significantly reduced DVT formation in vivo [56]. Overall, these findings indicate that thrombus initiation under reduced flow depends on the integrated actions of neutrophils, monocytes, platelets, endothelial cell activation and cell-derived tissue factor (Figure 2).

In this context, clonal hematopoiesis may be conceptualized not merely as a premalignant state, but as an active amplifier of chronic, low-grade inflammation and immunothrombotic priming, particularly in aging populations [4]. Somatic mutations in genes such as *TET2*, *DNMT3A*, and *ASXL1* reprogram myeloid cells toward a pro-inflammatory phenotype characterized by increased production of IL-1β, IL-6, and TNF-α [4]. This persistent cytokine signaling promotes endothelial cell activation, upregulation of adhesion molecules, oxidative stress, and enhanced platelet reactivity; all hallmarks of thrombo-inflammatory priming.

In parallel, chronic inflammation in aging (also known as “inflammaging”) itself creates a permissive microenvironment in which normal hematopoietic stem cells are functionally impaired, whereas mutant clones, which are resistant to inflammation, may gain a selective advantage, thereby reinforcing clonal expansion and sustaining systemic inflammation [4,57]. Inflammaging is characterized by elevated levels of blood inflammatory markers and is associated with a high susceptibility to chronic morbidity (particularly CVD), disability, frailty, and premature death [58]. This reciprocal interaction between clonal hematopoiesis and chronic inflammation may contribute to a persistent prothrombotic state, although the extent to which individual components of this pathway are directly mediated by CHIP in humans remains to be fully defined.

## 5. The Impact of Individual Somatic CHIP Mutations

The vascular and thromboinflammatory consequences of CHIP appear to differ substantially according to the underlying driver mutation. Current evidence suggests a hierarchical pattern in which *JAK2* V617F mutations confer the strongest overall thrombotic risk, particularly for venous thromboembolism, whereas *TET2* mutations have the most robust mechanistic support linking clonal hematopoiesis to inflammation-driven vascular disease. In contrast, *DNMT3A* and *ASXL1* mutations are consistently associated with clonal expansion and inflammatory signaling but have more modest and less well-defined effects on thrombotic risk in human studies. Importantly, the strength of evidence varies across endpoints, with arterial disease supported by more extensive and consistent data than venous thrombosis. This distinction underscores the need to interpret mutation-specific effects within the context of both vascular bed and level of supporting evidence. The following sections review the principal CHIP-associated mutations in relation to arterial disease, venous thrombosis, and the strength of evidence supporting mutation-specific thromboinflammatory mechanisms.

### 5.1. TET2 Mutations

*TET2* (Tet methylcytosine dioxygenase 2), a tumor suppressor gene, encodes a crucial enzyme that regulates DNA demethylation, essential for gene expression and hematopoietic stem cell differentiation. *TET2* is one of the most frequently mutated genes in clonal hematopoiesis and in hematologic malignancies. The regulatory influence of *TET2* on cell proliferation and apoptosis constitutes a central aspect of its biological function. Most CHIP-associated mutations result in loss of *TET2* function, leading to altered epigenetic regulation of gene expression and expansion of mutant hematopoietic clones. Through its role in DNA demethylation, *TET2* modulates transcriptional programs involved in cell differentiation, proliferation, and immune regulation. In particular, emerging evidence indicates that *TET2* plays a critical role in restraining inflammatory signaling in myeloid cells, positioning it as a key link between clonal hematopoiesis, chronic inflammation, and vascular disease [65,66]. Furthermore, the demethylation of DNA has implications in several aspects of cellular regulation and differentiation as well as in metabolic pathways, as seen from studies linking *TET2* activity to glucose tolerance and insulin resistance [67,68].

Emerging evidence indicates that loss of *TET2* function significantly impairs endothelial angiogenic capacity through the downregulation of key target genes within the STAT3 signaling pathway, a critical regulator of endothelial cell proliferation, survival, and vascular integrity. Since most CHIP-associated mutations result in reduced or absent TET2 activity, experimental studies have primarily relied on *TET2*-deficient models to investigate its biological consequences. In this context, Jaiswal et al. demonstrated that deletion of *TET2* in hematopoietic stem cells significantly exacerbates atherosclerotic plaque formation in susceptible mouse models [3]. Because human CHIP typically involves a specific percentage of mutant hematopoietic stem cells, subsequent studies in mice with knockout of the *TET2* gene used mixed chimerism to better mimic the human condition [3,59]. Similarly, these studies showed that even a small fraction of *TET2*-deficient cells is sufficient to increase the atheroma burden.

**Figure 3:**
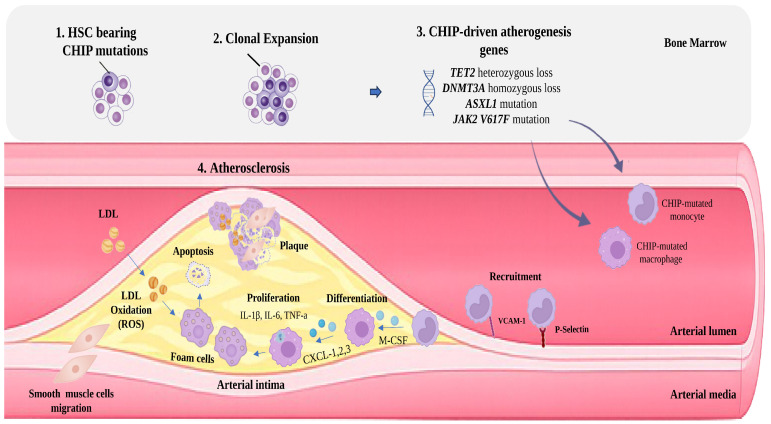
CHIP and atherosclerosis. HSCs with CHIP mutations undergo clonal expansion in the bone marrow, generating mutant myeloid cells (*TET2*, *DNMT3A*, *ASXL1*, *JAK2*) (1–3). CHIP-mutant monocytes are recruited to activated endothelium via adhesion molecules including VCAM-1 and P-selectin and infiltrate the arterial intima, where M-CSF drives their differentiation into macrophages (4). Macrophages release pro-inflammatory cytokines (IL-1β, IL-6, TNF-α) and chemokines (CXCL1-3), supporting local macrophage proliferation and leukocyte recruitment. Within the atherosclerotic lesion, oxidative stress and ROS promote LDL oxidation and uptake by macrophages, resulting in lipid accumulation and foam-cell formation. Lipid-laden foam cells undergo apoptosis, contributing to necrotic core formation and plaque expansion, while smooth muscle cells migrate into the intima and participate in plaque remodeling. CHIP, clonal hematopoiesis of indeterminate potential; CXCL, C-X-C motif chemokine ligand; HSC, hematopoietic stem cell; IL, interleukin; LDL, low-density lipoprotein; M-CSF, macrophage colony-stimulating factor; ROS, reactive oxygen species; TNF-α, tumor necrosis factor-α; VCAM-1, vascular cell adhesion molecule-1.

Across these models, *TET2* loss consistently drives a pro-inflammatory plaque milieu dominated by IL-1β, largely produced by *TET2*-mutant macrophages [3,59]. Selective deletion of TET2 in a subset of macrophages reproduces the accelerated plaque phenotype, underscoring their central role in disease progression [59]. Elevated cytokine output from *TET2*-mutant macrophages has been validated in additional studies and in a non-human primate model [69,70], identifying IL-1β–driven inflammation as a key mediator linking *TET2* deficiency to accelerated atherogenesis (Figure 3).

IL-1β is a central mediator of innate immune inflammation and is produced primarily by cells of the monocyte-macrophage system following activation of the NLRP3 inflammasome [59]. In clonal hematopoiesis driven by *TET2* mutations, this pathway becomes dysregulated, resulting in persistently elevated IL-1β levels and a background of chronic, smoldering inflammation [3]. This inflammatory state sensitizes the vascular endothelium to additional stimuli encountered during the early stages of thrombogenesis, such as extracellular nucleic acids, reactive oxygen species, and other inflammatory mediators [71,72,73]. In this setting, release of IL-1β and IL-18 by these infiltrating leukocytes stimulates endothelial IL-1 signaling, driving the expression of adhesion molecules and pro-coagulant mediators that favor platelet tethering and activation. Platelets themselves participate in a self-reinforcing loop: engagement of TLR4 and downstream BTK signaling activates platelet NLRP3, promoting IL-1 release and aggregation [5]. In parallel, vascular injury triggers abundant IL-1α release, which acts as an alarmin to maintain a pro-inflammatory, pro-thrombotic environment [71].

Downstream of these cytokine-driven events, inflammasome activation in macrophages leads to gasdermin D–dependent pyroptosis and liberation of tissue factor, setting off the coagulation cascade with robust thrombin generation and fibrin deposition. Neutrophil NLRP3 activation initiates PAD4-mediated NETosis, and the resulting extracellular traps perpetuate IL-1 signaling, enhance factor XII activation, and undermine natural anticoagulant pathways, including TFPI and antithrombin III. The DNA-rich backbone of NETs binds von Willebrand factor and fibrin, forming a durable scaffold that resists fibrinolysis and supports progressive thrombus growth. Together, these interconnected processes indicate that IL-1β–driven inflammasome activation may be the central mechanistic link between TET2-mutant clonal hematopoiesis and increased arterial and a plausible mechanism for venous thrombosis, warranting further experimental investigation [71,72,73].

### 5.2. DNMT3A Mutations

*DNMT3A* (DNA methyltransferase 3 alpha), a tumor suppressor gene, encodes a key enzyme that catalyzes DNA methylation, adding methyl groups to CpG islands to regulate gene expression. *DNMT3A* is the most frequently mutated gene in CHIP and is also commonly observed in AML, MDS, and other hematologic malignancies [74]. Similar to the *TET2* gene, loss-of-function mutations in the *DNMT3A* gene are also associated with atherosclerotic cardiovascular disease. After Jaiswal’s pivotal study in murine models [3], Rauch et al. [60] proceeded to a similar experiment with the *DNMT3A* mutation. More specifically, they studied mice transplanted with bone marrow from *DNMT3A* +/+ wild type (WT) mice, or from *DNMT3A* −/− knockout (KO) mice and WT mice in a 1:9 ratio to mimic the typical VAF of 10% seen in human CHIP, and fed them with a high-fat diet for several weeks. The researchers found that the mice with the *DNMT3A* mutation had a notable difference in the development of atherosclerotic plaque. Atherosclerosis occurs through inflammatory reprogramming of macrophages. RNA sequencing in *DNMT3A*-deficient bone marrow-derived macrophages revealed increased expression of CXC chemokines (CXCL1, CXCL2, CXCL3) and proinflammatory cytokines (IL-1β, IL-6) [60]. However, it should be noted that mutations in *DNMT3A* seem to have a weaker potency of inflammation as compared with *TET2* mutations. This is supported by the fact that *DNMT3A*-driven acceleration of atherosclerosis required homozygous loss, whereas *TET2* heterozygous loss was sufficient to promote plaque expansion. This distinction highlights a dosage-dependent effect for *DNMT3A*, contrasting with the more powerful inflammatory potential of *TET2* loss [60,75].

In summary, the *DNMT3A* mutation serves as a moderately potent proinflammatory factor and therefore its association with atherosclerosis is weaker than *TET2*. Regarding the venous branch of the vascular system, the data is even more limited and constitutes an important field of research. However, the coexistence of additional aggravating factors, particularly inflammatory conditions such as sepsis, may unmask or amplify a modest thrombotic risk associated with DNMT3A-mutant CHIP.

### 5.3. ASXL1 Mutations

*ASXL1* (Additional Sex Combs-Like 1) is a tumor suppressor gene that acts as an epigenetic regulator, controlling chromatin remodeling and gene expression (e.g., HOX genes) essential for blood cell development. *ASXL1* is inactivated by mutation in various myeloid malignancies and is linked to poor prognosis. Its exact role remains unclear, but it has been reported that it acts as a ligand-dependent co-activator for the retinoic acid receptor, and is also involved in chromatin remodeling [76,77]. In contrast to *TET2*- and *DNMT3A*-driven inflammatory programs, which are largely mediated through NLRP3 inflammasome activation, emerging evidence suggests that *ASXL1* mutations may preferentially engage alternative innate immune pathways. Yu et al. studied murine macrophages with truncating *ASXL1* mutations similar to those found in human CHIP [61]. Using CRISPR-edited hematopoietic stem cells, they produced bone-marrow–derived macrophages and examined their responses to inflammatory stimulation. The experiments demonstrated that *ASXL1* mutation in hematopoietic progenitor cells gives rise to macrophages with increased genomic instability, reflected by the accumulation of DNA damage, which in turn promotes selective activation of the AIM2 inflammasome. This leads to assembly of the AIM2–ASC–caspase-1 complex and enhanced maturation and secretion of the pro-inflammatory cytokines IL-1β and IL-6. These findings support a proinflammatory phenotype that may contribute to vascular inflammation and atherogenesis in the context of *ASXL1*-mutant clonal hematopoiesis.

Furthermore, *ASXL1*-mutant macrophages also exhibit enhanced expression and secretion of IL-10, an anti-inflammatory cytokine that mitigates cardiovascular risk, suggesting a compensatory regulatory mechanism aimed at counterbalancing AIM2-driven inflammation. This dual signaling profile highlights a more complex and potentially self-modulated inflammatory phenotype compared with other CHIP-associated mutations. Thus, AIM2 inflammasome activation represents a plausible pathway linking *ASXL1* mutations to vascular inflammation; however, in contrast to *TET2* and *JAK2* V617F, direct evidence linking *ASXL1* to thrombotic risk in humans remains limited [61,78].

### 5.4. JAK2 V617F Mutation

As discussed in the preceding section, DNMT3A and ASXL1 CHIP mutations promote atherosclerosis but show no significant association with venous thrombotic events, while TET2 mutations demonstrate a modest increase in VTE risk, which appears to depend on clone size. In contrast, CHIP carrying the JAK2 V617F mutation (often referred to as JAK2-CHIP) not only is associated with atherothrombotic cardiovascular disease but also contributes to VTE, conferring approximately a sixfold increase in VTE risk even in individuals with no evidence of a myeloproliferative phenotype [19,79]. Within this context, available data support distinct mechanisms underlying arterial and venous thrombosis.

Liu and colleagues combined large-scale human cohort meta-analysis with mouse models carrying only small *JAK2* V617F-mutant clones, and they demonstrated that even minimal mutant allele burdens (i.e., VAF as low as 1.5%) are capable of accelerating arterial clot formation and increasing platelet responsiveness [79]. Mutated megakaryocytes shifted platelet production toward a predominance of young, reticulated platelets with enhanced metabolic activity and a markedly prothrombotic profile. These newly generated platelets exhibited increased expression of cyclooxygenase (COX)-1 and COX-2, heightened cytosolic phospholipase A_2_ (cPLA_2_) activation, and excessive synthesis of thromboxane A_2_ (TXA_2_). Notably, TXA_2_ served not only to further stimulate *JAK2*-mutant platelets but also to activate neighbor wild-type platelets, creating a paracrine amplification loop that intensified thrombus formation. Collectively, the study findings establish platelet activation and TXA_2_-mediated platelet–platelet cross talk as central mechanisms linking *JAK2* V617F CHIP to arterial thrombosis [79]. Complementary studies suggest that this platelet-driven phenotype is reinforced by vascular inflammation and endothelial dysfunction. JAK2-mutant macrophages promote a pro-inflammatory vascular milieu that may favor thrombosis [80], while endothelial-specific expression of *JAK2* V617F has been shown to induce a prothrombotic state via increased P-selectin–mediated platelet adhesion [64].

In addition, experimental models of erosion-prone arterial lesions demonstrate that *JAK2* V617F-mutant hematopoiesis exacerbates endothelial injury, intimal apoptosis, and thrombus formation, in association with increased neutrophil extracellular trap (NET) formation and enhanced platelet recruitment [62]. Together, these findings support a model in which platelet activation, thromboxane-mediated amplification, vascular inflammation, endothelial dysfunction, and NET-driven thrombo-inflammation converge to promote arterial thrombosis in *JAK2*-mutant clonal hematopoiesis. However, it is important to note that these findings should be interpreted with caution, as several of these mechanisms derive from experimental systems or contexts with higher mutant burden and therefore, their direct applicability to CHIP—typically characterized by lower variant allele fractions and absence of overt hematologic disease—remains to be fully established.

In contrast to arterial thrombosis, where platelet-driven mechanisms predominate, venous thrombosis in the context of *JAK2*-CHIP appears to be more closely linked to leukocyte-mediated and coagulation-related pathways. Edelmann et al. [81] demonstrated that the *JAK2* V617F mutation promotes venous thrombosis through activation of β1/β2 integrins, resulting in enhanced leukocyte adhesion, endothelial interaction, and thrombus formation. This mechanism highlights the importance of cellular adhesion and immune–coagulative crosstalk in the venous system, where thrombus development is less dependent on high shear platelet activation and more reliant on interactions between leukocytes, endothelium, and the coagulation cascade.

In this context, the leukocytes recruited on the endothelial surface become active drivers of thrombus formation. *JAK2* V617F-mutant neutrophils show aberrant STAT3 and STAT5 signaling and increased reactive oxygen species (ROS) production, both of which facilitate PAD4-dependent chromatin decondensation, a key step in the extrusion of chromatin from neutrophils and formation of NETs. NETs provide a potent pro-thrombotic scaffold by activating the contact pathway of coagulation through factor XII, promoting platelet adhesion and aggregation, and inducing endothelial injury [63]. However, it is important to mention that evidence for enhanced NETosis in *JAK2*-mutant states is largely derived from ex vivo studies of neutrophils from patients with MPNs and MDS, and may not fully translate to *JAK2*-mutant CHIP. Whether neutrophils from individuals with *JAK2*-CHIP (i.e., without a myeloproliferative phenotype) also demonstrate increased NET formation remains to be seen.

## 6. Rare Thrombotic Syndromes Associated with Clonal Hematopoiesis: Paroxysmal Nocturnal Hemoglobinuria (PNH) and VEXAS Syndrome

Although distinct from clonal hematopoiesis of indeterminate potential (CHIP), certain hematologic disorders driven by somatic mutations could provide important insights into the relationship between clonal hematopoiesis, inflammation, and thrombosis. Paroxysmal nocturnal hemoglobinuria (PNH) and VEXAS syndrome are representative examples in which mutation-driven immune dysregulation is strongly linked to thrombotic complications. These conditions are discussed here as illustrative models of mutation-associated thromboinflammation and as conceptual frameworks that may help inform the investigation of underlying mechanisms of thrombosis in CHIP.

### 6.1. PNH

PNH is a clonal hematopoietic stem cell disorder caused by acquired somatic mutations in the phosphatidylinositol glycan class A (PIG-A) gene, which leads to loss of glycosylphosphatidylinositol (GPI)-anchored proteins on the cell membrane, including the complement regulators CD55 and CD59 [82]. This defect exposes erythrocytes to unrestrained complement attack, leading to chronic intravascular hemolysis, hemoglobinuria, nitric oxide (NO) depletion and a constellation of downstream complications. Τhe most clinically significant complication of PNH is life-threatening thrombosis, especially venous thrombotic events occurring in atypical sites such as hepatic, portal, and cerebral veins [82,83]. PNH has been referred to as “*the most vicious acquired thrombophilic state known in medicine*” [84]. In a recent multicenter study, it was reported that 39% of the patients experienced at least one thromboembolic event, confirming that thrombosis affects more than one-third of patients and remains a major clinical complication [85]. Data from the International PNH Registry confirm a clear dose–response relationship: thrombosis occurred in 4.6% of patients with clone sizes < 10%, 6.5% in those with 10–50%, and 17.6% among individuals with clone sizes > 50%. These findings underscore clone size as an important clinical biomarker of thrombotic risk [86]. While the clinical association between PNH and thrombosis is well established, the pathophysiological mechanisms underlying thrombotic complications of PNH remain incompletely defined. Several pathophysiologic hypotheses have been proposed, and an increasing number of studies continue to explore the molecular and cellular pathways that may explain this complex and multifactorial complication. The major proposed mechanisms underlying thrombosis in PNH include complement-mediated platelet activation, hemolysis-driven endothelial dysfunction, microparticle generation, impaired fibrinolysis, and complement–coagulation crosstalk [87].

Specifically, complement activation induces platelet activation, aggregation, and phosphatidylserine exposure, facilitating prothrombinase and tenase complex formation. Activated platelets release procoagulant microparticles (PMPs) expressing glycoproteins GPIb, GPIIb/IIIa, platelet endothelial cell adhesion molecule-1 (PECAM-1), and P-selectin, which can independently generate thrombin [88]. In addition, deposition of terminal C5b–9 complement complexes on platelets induces platelet activation and granule secretion which increases affinity for coagulation factors [89,90]. For example, ADP and serotonin are released from platelet dense granules on activation, and recent studies demonstrate that complement-induced ADP release activates both platelets and endothelial cells, underscoring the pivotal role of terminal complement activation in PNH-associated thrombosis [91].

Furthermore, intravascular hemolysis promotes thrombosis through free hemoglobin which depletes NO and releases heme and iron, generating reactive oxygen species (ROS) [92,93]. These oxidative pathways via excess ROS, heme, and platelet-derived signals induce endothelial injury and stimulate NET formation that supports fibrin deposition and coagulation [94]. In addition, NO depletion causes vasoconstriction, smooth muscle spasm, and platelet hyperreactivity and induces expression of P-selectin, VCAM-1, ICAM-1, and E-selectin by endothelial cells, promoting leukocyte recruitment [40,92]. Hemolysis also yields red cell–derived microvesicles that deliver heme to endothelial and immune cells, inducing tissue factor expression and amplifying coagulation [95].

Beyond hemolysis, PNH is characterized by deficiencies in GPI-anchored proteins involved in fibrinolysis and anticoagulation. Reduced urokinase plasminogen activator surface receptor (uPAR) impairs plasmin generation, whereas deficiency of GPI-bound TFPI weakens the natural inhibition of tissue factor–initiated coagulation, contributing to the persistence and propagation of thrombi [96,97].

Complement activation in PNH has been suggested to operate within a self-reinforcing vicious cycle. Blockade of C5 with eculizumab interrupts this cycle and leads to a marked reduction in thrombotic events, underscoring the central role of terminal complement activation in PNH-associated thrombogenesis [98]. While these mechanisms have been primarily described for PNH, they illustrate how somatic mutations in hematopoietic cells can drive thrombosis through immune-mediated pathways, a concept that may have broader relevance to clonal hematopoiesis.

### 6.2. VEXAS Syndrome

Vacuoles, E1 enzyme, X-linked, autoinflammatory, somatic (VEXAS) syndrome is a recently described clonal inflammatory disorder that is characterized by a heterogeneous spectrum of systemic autoinflammatory manifestations accompanied by progressive hematologic abnormalities, frequently fulfilling diagnostic criteria for myelodysplastic syndromes (MDS) or plasma cell dyscrasias. Beck et al. [99] utilized a genotype-first approach using exome sequencing in patients with severe, unexplained adult-onset inflammatory syndromes and identified recurrent somatic mutations in *UBA1*, the X-linked gene encoding the E1 enzyme, the major enzyme that initiates ubiquitylation. These mutations were restricted to hematopoietic stem and progenitor cells, thereby demonstrating a clonal hematopoietic origin of the disease. VEXAS syndrome is associated with a markedly increased risk of VTE and, thus, VEXAS syndrome may represent another mechanism of mutation-driven thromboinflammation.

Somatic mutations affecting methionine-41 (p.Met41) in *UBA1* disrupt the ubiquitination cascade, a pathway responsible for targeted protein turnover and cellular homeostasis. Impaired ubiquitin activation results in defective protein degradation, accumulation of misfolded or damaged proteins, and heightened cellular stress responses. This proteostatic imbalance subsequently triggers activation of innate immune signaling pathways, fostering a sustained proinflammatory state [99]. *UBA1* mutations often coexist with CHIP mutations in *DNMT3A*, *TET2*, or *ASXL1* genes. Screening for *UBA1* mutations should be considered in patients diagnosed with MDS, including those with unclassifiable MDS or MDS/MPN overlap syndromes [100]. Testing is particularly warranted when hematologic abnormalities coexist with prominent inflammatory features, such as recurrent fever, relapsing polychondritis, cutaneous vasculitis, or Sweet syndrome [101,102].

Notably, VEXAS syndrome is characterized by a significantly high risk of thrombotic events at a rate higher than 40% [101]. In a large multicenter retrospective cohort of 119 patients, Kusne et al. [101] showed that 58 patients (49%) had at least one thrombotic event, either VTE or arterial thrombosis, with venous thromboembolism (VTE) representing the dominant phenotype while arterial events developed less frequently but tended to accumulate progressively over time. Cumulative incidence analyses demonstrated that VTE risk is both early and sustained, reaching 17% at 1 year and 40% at 5 years from disease onset, whereas arterial thrombosis accumulated more gradually (6% at 1 year and 11% at 5 years). Importantly, 41% of VTE episodes were recurrent, nearly two-thirds were unprovoked, and 20% occurred despite therapeutic anticoagulation.

The mechanism by which mutations in *UBA1* cause thrombosis is not completely understood. It appears that loss of ubiquitination due to *UBA1* loss-of-function mutations leads to impaired protein turnover and intracellular stress which is morphologically seen as cytoplasmic vacuolization of immature myeloid and erythroid cells. Intracellular stress results in activation of innate immune signaling pathways and excessive production of proinflammatory cytokines by granulocytic cells. Markedly elevated factor VIII levels are observed in over 90% of patients, and increased von Willebrand factor levels in approximately 40%, supporting the concept of excessive activation of coagulation. Transcriptomic analyses have shown upregulation of TNF-α, IL-6, and interferon pathways, along with enhanced spontaneous NETosis and increased tissue factor expression by neutrophils and monocytes in VEXAS syndrome [101,103]. Lupus anticoagulant (LA) is detected in nearly 40% of cases, typically in the absence of anticardiolipin or β2-glycoprotein I antibodies, and correlates with elevated C-reactive protein levels. However, LA positivity may in fact reflect in vitro inflammation-related LA assay interference rather than representing a true antiphospholipid syndrome [101]. Additionally, venous inflammation (vasculitis) may contribute to thromboembolism in patients with VEXAS syndrome, through chronic myeloid-driven inflammation causing endothelial-cell dysfunction, similar to Behçet’s syndrome [104].

VEXAS syndrome is the prototype of mutation-driven autoinflammatory syndromes. Based on current evidence, it emerges that somatic mutations restricted to hematopoietic cells can induce systemic inflammatory activation and promote thrombosis, even in the absence of overt hematologic malignancy.

## 7. Discussion

Clonal hematopoiesis (CH) has emerged as a significant contributor to thrombotic disease, extending far beyond its initial designation as an age-related phenomenon. Accumulating epidemiologic data support an association between CHIP and arterial thrombotic events, while more recent studies suggest a more modest and heterogeneous relationship with venous thromboembolism. These findings highlight the complexity of mutation-driven vascular risk and underscore the need to interpret CHIP not as a uniform entity, but as a biologically diverse condition with gene-specific effects. Available data support that carriers of *TET2*, *DNMT3A*, *ASXL1*, or *JAK2* mutations have a markedly increased risk of myocardial infarction, stroke, and cardiovascular mortality [2,3,14]. Regarding VTE risk, ARIC, UK Biobank, and case–control analyses show a modest but reproducible elevation in VTE incidence among CHIP carriers, particularly those with *TET2* or *JAK2* mutations. These findings suggest that screening for CHIP mutations may represent a relevant modifier of thrombotic risk in selected patients with otherwise unexplained coronary ischemic events as well as patients with other undiagnosed arterial or venous thrombotic events. There may also be a role for CHIP in cancer-related thrombosis, especially in patients 50 years of age or older, although current evidence remains limited.

The COVID-19 pandemic has brought immunothrombosis into global focus, demonstrating how dysregulated innate immunity—particularly IL-1, IL-6, and interferon signaling—can precipitate diffuse endothelial injury and thrombosis [6,48]. This recognition has broadened our understanding of inflammatory thrombosis and highlighted the need for more human-based research, rather than relying solely on murine models. In this context, insights from COVID-19–associated coagulopathy have provided a valuable framework for investigating the pathogenesis of immunothrombosis, supporting the biological plausibility of inflammation-driven endothelial dysfunction in CHIP-associated thrombotic disease, although direct CHIP-specific evidence remains limited.

Growing evidence indicates that the thrombotic consequences of CHIP are likely mediated through an immunothrombotic framework, in which inflammatory and hemostatic pathways become pathologically connected. CHIP-related mutations promote a low-grade inflammatory milieu (“inflammaging”), endothelial activation, and monocyte-driven tissue factor expression and have been associated with enhanced NETosis, platelet priming, and release of von Willebrand factor [4,12,57]. Disease models such as PNH and VEXAS syndrome illustrate aspects of this paradigm: PNH demonstrates how complement-driven platelet activation and hemolysis produce thrombosis, whereas VEXAS syndrome shows how *UBA1*-mutant clones drive extreme cytokinemia, NETosis, high factor VIII, and vasculitic endotheliopathy leading to a >40% VTE incidence. Together, these observations support a proposed feed-forward loop in which clonal myeloid cells amplify inflammation, inflammation accelerates clonal expansion, and both jointly promote thrombosis. While parallels between arterial and venous pathways are evident, CHIP should be considered a potential unifying contributor to thromboinflammation rather than a definitively established driver across vascular beds.

The relationship between clonal hematopoiesis and thrombosis is not uniform across all genes but is dependent on clone size (as measured by VAF) and specific mutation, i.e., it is a dose-dependent and mutation-specific relationship. In age-related CHIP involving *TET2*, *DNMT3A*, and *ASXL1* genes, several large cohort studies have demonstrated that expanding clone size is associated with a stepwise increase in thrombotic risk, with VAF ≥ 10% marking a threshold beyond which venous and arterial events become more frequent [19,22]. This pattern mirrors the well-described clone–thrombosis gradient observed in PNH, where larger GPI-deficient clones confer markedly higher rates of life-threatening venous thrombosis [85]. In contrast, some mutations exert a strong prothrombotic influence that may be less dependent on VAF magnitude. *JAK2*-mutant CHIP, for example, is associated with a disproportionately elevated risk for VTE and arterial thrombosis even at small clonal fractions, suggesting that its intrinsic myeloproliferative and proinflammatory signaling—rather than clone size alone—may contribute substantially to thrombogenesis [19,22,79]. Similarly, in VEXAS syndrome caused by *UBA1* mutations, thrombotic risk appears to correlate more closely with systemic inflammation, cytokine excess, and inflammation of vessel walls (vasculitis) rather than with the mutant allele fraction [101,102,103]. Overall, current evidence indicates that thrombotic risk in clonal hematopoiesis is shaped by both quantitative burden and qualitative mutation biology, with clone size serving as an important modifier in some contexts but not as the sole determinant of risk.

These findings carry important clinical implications. Next-generation sequencing (NGS) may offer additional insights into VTE risk stratification, particularly in older individuals, in those with recurrent or unprovoked VTE, or in patients with comorbid inflammatory or cardiovascular disease. Understanding an individual’s mutational profile, including gene, VAF, and co-mutational architecture, may help refine prognosis and guide personalized prevention strategies. The inflammatory basis of thrombosis raises the possibility of targeted anti-inflammatory interventions: the CANTOS trial demonstrated that IL-1β inhibition can reduce cardiovascular events [73], providing a proof-of-concept framework for future studies of CHIP carriers.

Collectively, available evidence supports a conceptual framework in which mutation-driven innate immune dysregulation promotes endothelial activation, platelet stimulation, and thrombin generation, linking clonal hematopoiesis to thromboinflammation. Observations from related clonal disorders such as PNH and VEXAS provide additional context for how distinct somatic mutations can drive similar thromboinflammatory phenotypes through different biological pathways. Future studies integrating genomic profiling with prospective clinical outcomes will be essential to clarify the causal role of CHIP in thrombosis and to determine whether it can be incorporated into risk stratification models or targeted therapeutically. A better understanding of mutation-specific effects, clonal dynamics, and interactions with environmental risk factors will be critical for translating these insights into clinical practice.

## 8. Conclusions

Available data support a conceptual framework in which clonal hematopoiesis contributes to thrombotic disease through dysregulated innate immune signaling and its downstream effects on endothelial activation, platelet function, and coagulation. However, the strength of evidence varies across mutations and clinical settings, with *JAK2* V617F demonstrating the most consistent thrombotic signal and *TET2* providing the strongest mechanistic link to inflammation, while other mutations remain less clearly defined. Importantly, several aspects of this model are derived from experimental systems and related clonal disorders, and direct CHIP-specific human evidence remains limited for key pathways, particularly in venous thrombosis.

From a clinical perspective, CHIP should be viewed not only as a risk factor for the development and progression of hematologic cancer but also as a potential modifier of an individual’s thrombotic risk. While its identification may provide additional insight into selected patients such as those with unexplained or recurrent thrombotic events, the routine use of CHIP for risk stratification or screening is not yet supported by sufficient evidence. Nevertheless, the integration of genomic data with inflammatory and clinical parameters represents a promising avenue for future research, with the potential to refine risk prediction and guide targeted preventive strategies. As the field advances, we expect to see more studies on CHIP that could lead to important gains in understanding the pathogenesis of both leukemogenesis and thromboinflammation.

## Figures and Tables

**Table 1:** Key differences between myelodysplastic syndromes and “pre-myelodysplastic” conditions.

	ICUS *	CHIP †	CCUS ‡	MDS
Clonality	−	+	+	+
Dysplasia	−	−	−	+
Cytopenia	+	−	+	+
Blasts (marrow)	<5%	<5%	<5%	0–19%
Cytogenetic abnormalities	−	+/−	−	+
Molecular deviations	−	+	+	++
Number of mutated genes	0	1–2	1–3	≥2
VAF	0	2–10%	variable	>20%
Risk of myeloid malignancy/year	<1%	<1%	LR < 1% HR~10%	−

* ICUS is defined as the presence of cytopenia in one or more hematological lineages (red blood cells, neutrophils, or platelets) that persists for ≥4 months, does not meet the diagnostic criteria for MDS, and is not attributable to any other hematological or non-hematological disease. Patients are further subdivided into ICUS-A (anemia), ICUS-N (neutropenia), ICUS-T (thrombocytopenia), and ICUS-PAN (pancytopenia) according to the type and number of cytopenias. † CHIP is defined as the detection of somatic mutations in genes associated with myeloid malignancies in peripheral blood or bone marrow at a VAF ≥ 2% (≥4% for X-linked genes in males), in individuals without a previously diagnosed hematological disease or unexplained cytopenia. ‡ CCUS is defined as the acquisition of a clonal lesion in patients with cytopenia who do not fulfill the minimum diagnostic criteria for MDS and in whom no other hematological neoplasm is detected. ICUS: idiopathic cytopenia of undetermined significance, CHIP: clonal hematopoiesis of indeterminate potential, CCUS: clonal cytopenia of unknown significance, MDS: myelodysplastic syndrome, VAF: variant allele frequency, LR: low risk, HR: high risk.

**Table 2:** Summary of key epidemiologic studies evaluating the association between CHIP and arterial and venous thrombosis.

Study (Year) Ref	Design and Study Population	Gene	Arterial Versus Venous Endpoint	Clone-Size	Effect Estimate	Key Limitations
Jaiswal (2014) [2]	Prospective cohort (population-based study) (*n* = 17,182)	Any CHIP driver mutation	Arterial (CAD, stroke)	VAF ≥ 2%	CAD HR 1.9–2.0 Ischemic stroke HR 2.6	Landmark study; mixed cohorts; not gene-specific analysis
Jaiswal (2017) [3]	Prospective cohort (case–control study)	*DNMT3A* *TET2* *ASXL1* *JAK2 V617F*	Arterial (CAD, stroke)	VAF > 10% (large clone)	*DNMT3A/TET2/ASXL1*: HR~1.7–2.0 (CAD) *JAK2 V617F*: HR~12.1 (CAD) Large clone: HR 2.2 versus HR 1.4	Mixed study designs; limited gene-specific precision (small subgroup sizes)
Bick (2020) [11]	Prospective cohort UK Biobank data	*DNMT3A* *TET2*	Arterial (MI, revascularization, stroke, or death)	VAF ≥ 10% (large clone)	Any CHIP HR 1.27 Large clone HR 1.59	Composite endpoint includes all-cause death; focused on *DNMT3A/TET2* genes
Vlasschaert (2023) [12]	Prospective cohort UK Biobank data (*n* = 451,180)	Any CHIP driver mutation	Arterial (CAD)	VAF ≥ 10% (large clone)	Any CHIP: HR 1.22 (95% CI 1.12–1.32); Large clone (VAF ≥ 10%): HR 1.25 (95% CI 1.13–1.39); IL6R protective for large clones	Brief report; gene-specific HR not reported
Bhattacharya (2022) [14]	Prospective cohort 8 biobanks/cohorts data	Any CHIP driver mutation	Arterial (Stroke)	VAF ≥ 10%	Overall stroke: HR 1.4–1.5 *TET2*: strongest association	Stroke subtyping reduces events statistical power; *TET2* HR not provided in text
Marston (2024) [15]	RCT substudy (TIMI trials) (*n* = 63,700)	Any CHIP driver mutation	Arterial (MACE, MI)	Not reported	Overall MACE: HR 1.07 First MI: HR 1.31 Recurrent MI: not significant	RCT population under intensive secondary prevention therapy; limited to post-MI cohort results may not apply to the general population.
Saadatagah (2025) [16,17]	Prospective cohort ARIC study	Any CHIP driver mutation	Venous (VTE)	VAF ≥ 2%	Any CHIP HR 1.49 (95% CI 1.02–2.17) *TET2* HR 2.25	Moderate sample size and limited number of VTE events; possible residual confounding from occult malignancy.
Dikilitas (2021) [18]	Prospective cohort UK Biobank data	Any CHIP driver mutation	Venous (VTE, PE)	Not reported	VTE IRR 1.60 (95% CI 1.04–2.46) PE IRR 1.80 (95% CI 1.08–3.05), *TET2*: strongest association	Published as abstract only
Zon (2024) [19]	Prospective cohort (cross-sectional analysis) (*n* ≈ 400,000)	Any CHIP *TET2* *JAK2 V617F* *DNMT3A* *ASXL1*	Venous (VTE)	VAF ≥ 10% (large-clone)	Any CHIP: incident HR 1.17, VAF ≥ 10%: incident HR 1.23 *TET2*: HR 1.33 *JAK2 V617F*: incident HR 4.2, prevalent OR 6.58 *JAK2 V617F*: incident HR 6.24, prevalent OR 11.88 *DNMT3A/ASXL1*: not significant	WES may under-detect small *JAK2 V617F* clones; possible MPN misclassification as CHIP
Englisch (2025) [20]	Case–control study	Any CHIP driver mutation	Venous (VTE)	Not reported	CHIP in 10.3% cases versus 3.9% controls OR 2.74 (95% CI 0.95–9.16)	Borderline significance (wide CI); small sample; published as abstract only
Soudet (2021) [21]	Retrospective cohort (case–control study) (*n* = 61)	Any CHIP driver mutation	Venous (PE)	No VAF threshold reported	CHIP in 19.7% of unprovoked PE versus matched controls	Small sample study; no adjusted effect estimate (OR, HR) provided
Liu (2024) [22]	Prospective cohort UK Biobank data (*n* = 464,417)	Any CHIP *TET2* *JAK2 V617F* *DNMT3A* *ASXL1* *PPM1D* *SRSF2*	Venous (PE)	VAF ≥ 2%	Any CHIP HR 1.17 (95% CI 1.05–1.31) *TET2* HR 1.42 (95% CI 1.16–1.74) *JAK2 V617F* HR 4.17 (95% CI 2.09–8.35) *DNMT3A/ASXL1/* *PPM1D/SRSF2*: not significant	PE-specific endpoint rather than all-VTE; observational design
Svensson (2022) [23]	RCT substudy CANTOS trial	*DNMT3A* *TET2*	Arterial (CAD, secondary MACE)	No VAF threshold reported	Placebo CHIP: HR 1.32 *TET2* + canakinumab: HR 0.38	Post hoc analysis; limited to post-MI cohort

CH, clonal hematopoiesis; CHIP, clonal hematopoiesis of indeterminate potential; CI, confidence interval; HR, hazard ratio; IRR, incidence rate ratio; OR, odds ratio; PE, pulmonary embolism; VTE, venous thromboembolism; MACE, major adverse cardiovascular events; CAD, coronary artery disease; PAD, peripheral artery disease; VAF, variant allele frequency; MPN, myeloproliferative neoplasm; WES, whole-exome sequencing.

## Data Availability

This is a review article with no new data created.
